# Molecular Determinants of Ethionamide Resistance in Clinical Isolates of *Mycobacterium tuberculosis*

**DOI:** 10.3390/antibiotics11020133

**Published:** 2022-01-20

**Authors:** Anastasia Ushtanit, Elena Kulagina, Yulia Mikhailova, Marina Makarova, Svetlana Safonova, Danila Zimenkov

**Affiliations:** 1 Center for Precision Genome Editing and Genetic Technologies for Biomedicine, Engelhardt Institute of Molecular Biology, Russian Academy of Sciences, 119991 Moscow, Russia; ushtanit@mail.ru (A.U.); elenka176@yandex.ru (E.K.); 2 The Moscow Research and Clinical Center for Tuberculosis Control, Moscow Government Health Department, 107014 Moscow, Russia; juliaisaeva81@rambler.ru (Y.M.); makarova75@yandex.ru (M.M.); safonova.s.g@inbox.ru (S.S.)

**Keywords:** tuberculosis, drug resistance, ethionamide, isoniazid, perchlozone

## Abstract

Background: Ethionamide and prothionamide are now included in group C of the WHO recommended drugs for the treatment of tuberculosis resistant to rifampicin and multidrug-resistant tuberculosis. The clinical relevance of ethionamide and prothionamide has increased with the wide spread of resistant tuberculosis. Methods: We retrospectively analyzed 349 clinical isolates obtained between 2016 and 2020. The susceptibility to ethionamide was tested using both the Bactec^TM^ MGIT^TM^ 960 system and the Sensititre^TM^ MYCOTB plate. Results: The MIC of ethionamide increases with the total resistance of the isolates in a row from susceptible to XDR strains. A significant part of the isolates have a MIC below the breakpoint: 25%, 36%, and 50% for XDR, pre-XDR, and MDR strains. Sensitivity and specificity of detection of mutations were 96% and 86% using MGIT resistance as a reference. Conclusions: Phenotypic methods for testing ethionamide are imperfectly correlated, and the isolates with MIC of 5 mg/L have the intermediate resistance. A significant proportion of resistant TB cases are susceptible and eligible for ethionamide treatment. Resistance could be explained using only analysis of loci *ethA*, P*_fabG1_*, and *inhA* for most isolates in the Moscow region. The promoter mutation P*_fabG1_* c(-15)t predicts resistance to ethionamide with high specificity but low sensitivity.

## 1. Introduction

Ethionamide (2-ethylthioisonicotinamide) was synthesized in 1952 by D. Liberman and from 1955 onward had limited use in the treatment of tuberculosis [1]. In 1956, a structurally similar prothionamide, with slightly lower efficacy but better tolerability, was discovered [2]. The clinical relevance of ethionamide and prothionamide has increased with the wide spread of resistant tuberculosis. With the successful introduction of new and repurposed drugs bedaquiline, linezolid, clofazimine, and delamanid into treatment regimens, ethionamide and prothionamide are now included in group C of the WHO recommended drugs for rifampicin-resistant and multidrug-resistant tuberculosis (MDR) treatment [3]. This group also includes pyrazinamide, ethambutol, amikacin, PAS, imipenem, meropenem, and streptomycin; these drugs should be prescribed if drugs from groups A and B must be excluded due to resistance or side effects. It can be assumed that ethionamide will not be completely eliminated from therapy schemes in the near future, given the high cost of new drugs and the emergence of resistant forms of these drugs as well [3].

**Figure 1:**
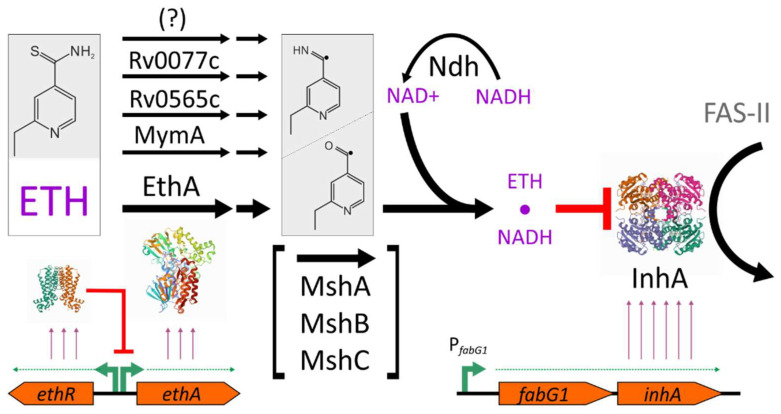
Model of ethionamide action on *M. tuberculosis*. Ethionamide is activated in a multistep process, mostly due to EthA monooxygenase activity. Ethionamide-NADH adduct inhibits the enoyl acyl carrier protein reductase InhA, catalyzing one of the steps in FAS-II cycle of mycolic acid biosynthesis. The transcription of the *ethA* gene is regulated by the EthR repressor. Mycothiol biosynthesis genes (*mshA*, *mshB*, *mshC*) or its product, mycothiol, are also involved in the ethionamide activation, while its particular roles are still not revealed. The protein structures shown are from the RCSB PDB database: EthR—5NJ0 [23], EthA homolog —3UOZ [24], and InhA—4TRN [25].

Ethionamide is a prodrug that requires bioactivation, which is also characteristic of other antituberculosis drugs: isoniazid, pyrazinamide, PAS, delamanid, and pretomanid. Ethionamide activation is a multistage process, while detailed mechanisms are still not fully revealed (Figure 1) [4]. The main enzyme that provides the conversion of ethionamide to active radicals, which are further converted to a toxic adduct with NADH, is Baeyer-Villiger monooxygenase EthA [5,6,7]. The majority of resistant clinical strains had alterations at the *ethA* locus; approximately one third of them are loss-of-function frameshift and nonsense mutations [8,9]. 

The resulting adduct of ethionamide and NAD+ inhibits the enoyl acyl carrier protein reductase InhA, an essential FAS-II enzyme of the cell wall mycolic acid synthesis cycle [10,11]. InhA is also a target of isoniazid, which also requires bioactivation to exhibit antituberculosis properties. The activator in the case of isoniazid is catalase/peroxidase KatG. Therefore, partial cross-resistance between ethionamide and isoniazid is observed in clinical isolates [12] due to *inhA* gene mutations, which affect its binding properties with toxic adducts. Alternatively, promoter mutations of the *inhA* gene that lead to an increased transcription rate result in an increased number of InhA target proteins in the cell, thus diminishing the toxic action of both drugs [8]. 

The expression of *ethA* is regulated by the divergently transcribed *ethR* repressor gene. Therefore, aminoacid substitutions in EthR, providing a more tight binding of the repressor, and thus lowering *ethA* transcription, could also be selected during treatment [9,13]. Other transcription affecting mutations located in the *ethA-ethR* intergenic region were associated with a moderate increase in MIC [14,15], and for one of them a lower *ethA* transcription rate was experimentally demonstrated [16]. More importantly, this mutation was selected independently by many sublineages and impacts the resistance to ethionamide synergistically with mutations in other loci [16].

In addition to EthA, there are five more predicted Baeyer-Villiger monooxygenases in *M. tuberculosis*: Rv0892, Rv0565c, Rv1393c, Rv3049c, and MymA (Rv3083) [17]. The ability to activate ethionamide has also been demonstrated for MymA and Rv0565c [18]. Mutations in these loci were detected in clinical strains; however, the questions about clinical significance and co-occurrence with *ethA* mutations are still open [19]. In another report, clinical strains with *mymA* gene deletion have only a minor increase in MIC far below the clinical breakpoint [16]. 

A possible role of oxidoreductase Rv0077c in ethionamide activation was discovered during the selection of molecules capable of reversing ethionamide resistance in *ethA*- strains. The specific inducer SMARt-420 binds to the Rv0078 repressor, leading to the expression of the *rv0077c* gene and the susceptible phenotype [20]. 

At least two more metabolic pathways are also involved in ethionamide activation. First, the NAD+:NADH ratio affects the conversion step of ethionamide-derived radicals to ethionamide-NADH adduct [21]. However, in a recent report, *ndh* mutations were only found in combination with *ethA* and *inhA* promoter mutations [9]. The second is a mycothiol biosynthesis pathway: while the exact mechanism of action has not yet been revealed, *msh*- strains have an Eth^R^ phenotype [22]. As with *ndh*, *mshA* mutations were identified only in a small proportion of clinical strains, in combination with *ethA* or *inhA* promoter mutations [9].

Our aim was to analyze the distribution of minimum inhibitory concentrations of ethionamide (MIC) for clinical isolates of *M. tuberculosis* circulating in the Moscow region, and to identify molecular determinants of reduced susceptibility of ethionamide. Taking into the account the low frequency of mutations located in *ndh*, *mshA*, and *ethR* genes in ethionamide-resistant clinical strains, the study settings were restricted to the analysis of key resistance determinants in *ethA*, *inhA*, *ethA-ethR* intergenic region and *inhA* promoter loci. Combining the phenotypic and molecular data, we estimated the proportion of drug-resistant isolates that are eligible for ethionamide treatment.

## 2. Results

### 2.1. The MIC of Ethionamide Depends on the Resistance Profile of the Isolate

We retrospectively analyzed 349 clinical isolates, obtained in 2016–2020 from patients who attended the Moscow Research and Clinical Center for Tuberculosis Control. The susceptibility to ethionamide was tested using both the Bactec^TM^ MGIT^TM^ 960 (MGIT) system (critical concentration of 5 mg/L) and the Sensititre^TM^ MYCOTB microtiter plate for MIC determination (critical concentration of 5 mg/L [MYCOTB]). An imperfect correlation of the binary resistant/susceptible (R/S) and MIC data were observed (Table 1). Forty-three percent of resistant isolates had MIC ≤ 5 mg/L and should therefore have been considered susceptible from the Sensititre data. Additionally, 6% of susceptible isolates had MIC ≥ 10 mg/L. 

The isolates studied were divided into groups based on their resistant profiles: susceptible, poly- or monoresistant, MDR, extensively drug-resistant (XDR) tuberculosis, and pre-XDR. Here we used the old definition of XDR tuberculosis, i.e., MDR plus resistance to any fluoroquinolone and to any of the injectable second-line drugs—kanamycin, amikacin, or capreomycin. Pre-XDR are two intermediate categories between MDR and XDR, when additional resistance to fluoroquinolones, or injectable drugs are registered but not both at once. Noticeably, 53 isolates susceptible to all drugs had a more compact MIC distribution compared to 176 isolates susceptible to ethionamide joined from all groups. Its mode of distribution was at a MIC of 2.5 mg/L, while MIC_90_ was equal to 5 mg/L. Following this finding, the lower breakpoint concentration of 2.5 mg/L for Sensititre assay could be proposed; however, no change of performance compared to MGIT data could be achieved. The MIC of ethionamide increases with the total resistance of isolates in a row from susceptible to XDR strains.

Based on the currently approved critical concentration of 5 mg/L for Sensititre assay, almost 50 % of XDR isolates are eligible for ethionamide treatment. For pre-XDR and MDR isolates, the proportion of presumably susceptible is raised to 63 and 69%, respectively. Even if the lower critical concentration of 2.5 mg/L is used, a significant part of the isolates have a MIC of ethionamide below the breakpoint: 25%, 36% and 50% for XDR, pre-XDR and MDR strains (Table 1).

As a reference to the data obtained, the isoniazid resistance of these isolates was also analyzed (Table S1). Isoniazid and ethionamide share common resistance determinants: substitutions in the *inhA* gene and its promoter mutations. However, the most frequent mutation in isoniazid-resistant isolates is the substitution of KatG S315T, so cross-resistance between these drugs is a rare event. It was confirmed by pairwise comparison of the MIC data (Table 2). Two distinct peaks at 0.06 mg/L and >4 mg/L were observed for the distribution of isoniazid MIC, separating susceptible and resistant isolates. Cross-resistance between two drugs was low, so a significant part of high level isoniazid-resistant strains were ethionamide-susceptible.

### 2.2. Molecular Determinants of Ethionamide Resistance

To study the molecular mechanisms of resistance to ethionamide, we selected only primary culture-positive tuberculosis cases, diagnosed in 2017–2018 (*n* = 136). We sequenced *ethA*, *inhA*, and *inhA* promoter region upstream the *fabG1* gene. Thirty-two isolates had a MIC of 10 mg/L or higher.

Mutations were found in 94% of resistant isolates. However, the detection specificity was approximately 74% (Table 3). Part of the identified mutations were aminoacid substitutions in the EthA protein, which have unknown impact on its activity (Table S2). Its exclusion from prediction increases the specificity to 89%, simultaneously lowering the sensitivity to 84%. Importantly, part of the frameshift and nonsense mutations in *ethA* did not lead to an increase in MIC (*n* = 7 from 19 with such type of mutations).

Promoter mutations in the *inhA* gene, located upstream of *fabG1*, were previously proposed to be a marker of resistance to ethionamide [26,27]. The most common mutation c(-15)t is usually accompanied by KatG S315T substitution in isoniazid-resistant strains. Taking into account the analyzed set of isolates, the sensitivity and specificity of this mutation in the prediction of ethionamide resistance were 31% and 98%, respectively (Table 3), which is close to previously reported sensitivities of 38% [28] and 43% [29]; however, in one study a sensitivity of 92% was reported [30].

The sensitivity and specificity of detection of any mutation (excluding synonymous) was 97% and 74% for resistance detected with 96-well plate data and 96% and 86% for Bactec MGIT resistance. Exclusion of EthA substitutions leads to decrease of sensitivity with simultaneous increase of specificity: 87% and 86%; 80% and 94%, for Sensititre and MGIT data, respectively.

## 3. Discussion

Mycolic acid biosynthesis is an attractive target for tuberculosis treatment. One of the first drugs, thiacetazone, developed in 1946 by Gerhard Domagk, inhibits one of the steps in the FAS-II cycle [31]. Although thiacetazone was excluded from treatment schemes due to toxicity and low efficiency, another drug, isoniazid, which inhibits the other step in the FAS-II cycle, remains one of the cornerstones for the treatment of active and latent tuberculosis. Ethionamide shares the target with isoniazid; however, the cross-resistance between them is not frequent. We analyzed the resitance of isoniazid and ethionamide for a large set of clinical isolates from the Moscow region with different resistance profiles. 

First, we found a poor correlation of the binary resistance (R/S) of ethionamide obtained with Bactec MGIT 960 and the determination of MIC with Sensititre MYCOTB. MIC ranges for susceptible and resistant isolates intersect significantly, which is in line with previous reports [32]. The cause of such a discrepancy of two methods that use the same liquid 7H9 medium remains unrevealed.

Compared to ethionamide, isoniazid showed a strict difference between susceptible and resistant isolates. Its modes of MIC distribution are separated in six or more dilution steps. Possible explanations for this phenomenon include the wide use of isoniazid as a first-line drug for TB treatment and the high level of transmission of MDR tuberculosis in Russia. We recently compared MDR strains from Russia and Taiwan [33]: while strains from Moscow had narrow spectra of molecular determinants of isoniazid resistance and high MIC, isolates from Taiwan possessed a wider range of MICs and variable spectra of drug-determining mutations reflecting ongoing selection.

Second, a lower critical concentration of ethionamide could be proposed for microdilution plates on the basis of the MIC ranges for susceptible isolates. Currently, the approved concentration is 5 mg/L, while most ethionamide naïve isolates have a MIC < 2.5 mg/L. This value also better fits previously published pharmacokinetic data [34,35,36]. However, the overlap of MIC distributions between resistant and susceptible isolates reflects only the ‘intermediate’ resistance of isolates with MIC = 5 mg/L, and further clinical studies of ethionamide treatment effectiveness in such cases are needed.

Most mutations leading to resistance to ethionamide in clinical strains are found in the *ethA* and *inhA* genes. Substitutions in the promoter region of the isoniazid and ethionamide target gene, *inhA*, leading to increased gene expression should lead to cross-resistance [12]. However, data on the association of the most common P*_fabG1_* c(-15)t mutation and resistance to ethionamide and prothionamide are inconsistent, as resistant and sensitive strains with this mutation have been found [9,37,38,39]. This substitution in the promoter is associated with an increase in the MIC of isoniazid to an intermediate level that cannot be unambiguously interpreted by critical concentration or proportion methods [40]. However, in the case of ethionamide, the variability in the stability level is more pronounced, with a difference of up to two orders of magnitude in the MIC concentration [37]. In our set of isolates, we found high specificity, close to 100%, and low sensitivity of detection of this mutation for the prediction of resistance to ethionamide.

We also found that frameshift and nonsense mutations in *ethA* gene do not necessarily lead to the loss of activation and resistance to ethionamide in bacterial cell: 37% of strains had MIC in susceptible range. It is consistent with previous studies, where 35% of the *ethA*-inactivating mutations of strains were susceptible to prothionamide [14]. It was previously reported that other monooxygenases are also active toward ethionamide. MymA activity levels in wild-type cells are comparable to EthA [18], and it appears that *mymA*-inactivating mutations can also be selected by ethionamide-based drug therapy [14]. However, it is not clear whether mutations in *ethA* in ethionamide-susceptible isolates were selected by the drug action, are the result of neutral evolution and genetic drift, or are just a drawback in currently used phenotypic methods. The latter could be explained by the selection of more tolerant, not resistant strains, i.e., a longer survival time at sub-inhibitory concentrations of the drug [41].

The correlation between genotype and ethionamide-resistant phenotype is still an open question because while mutations in the *ethA*, *mshA*, *ndh* and *inhA* loci were detected in 96.5% of cases [28], in most other studies mutations were not detected in a significant percentage of strains. For example, in a 2011 study, 8 of 47 isolates (17%) were found to have no mutations when analyzing *inhA*, *ethA/R*, *ndh*, *mshA* [13]. Similarly, a recent study found no mutations in 36 of 178 isolates (20%) when analyzing the same determinants [9]. Furthermore, when a wider range of loci (*inhA*, *ethA/R*, *ndh*, *mshA/B/C/D*) were analyzed, no mutations were found in 9 of 46 prothionamide resistant strains [42]. It should be noted that 74% of selected in vitro ethionamide-resistant strains had no mutations in *ethA/R, katG* and *inhA* also [43]. We analyze the loci *ethA*, *ethA-ethR*, *inhA*, and P*_fabG1_* and achieved 97% sensitivity with 74% specificity if MIC data were used with a critical concentration of 5 mg/L. Using the Bactec MGIT 960 system resistance data as reference, the specificity increased to 86%, while the sensitivity left unchanged. We suppose that the analysis of wider panel of molecular determinants would not lead to the increase in performance due to limitations in phenotypic methods observed in the study—24% overlap in MICs between susceptible and resistant isolates.

In conclusion, determination of ethionamide MIC before treatment is highly desirable, taking into account the inability to increase doses due to drug toxicity. Furthermore, a large individual variation of pK/pD parameters [36] and interaction with other drugs [35,44] require personalized treatment with therapeutic drug monitoring.

## 4. Materials and Methods

### 4.1. Mycobacterium Tuberculosis Strains

The *M. tuberculosis* strains were obtained from clinical specimens collected from TB patients at the Moscow Research and Clinical Center for Tuberculosis Control. In total, 349 clinical isolates, obtained in 2016–2020 from patients who attended the Moscow Research and Clinical Center for Tuberculosis Control were analyzed: 2016—50 isolates, 2017—87 isolates, 2018—66 isolates, 2019—74 isolates, and 202—72 isolates. For molecular analysis only primary isolates from 2017–2018 were used. They were isolated sequentally, one isolate for one patient, before treatment initiation. Drug susceptibility testing for rifampicin, isoniazid, streptomycin, ethambutol, pyrazinamide, ofloxacin, moxifloxacin, kanamycin, capreomycin, and amikacin, PAS, ethionamide was performed using Bactec MGIT 960 as previously described [45,46]. Sensititre MYCOTB MIC determination was performed as described in [47].

The study was approved by the Ethics Committee of the Moscow Government Health Department. The Ethics Committee waived the need for patient consent because the study did not include personal identifiers or clinical data and the samples were analyzed anonymously. 

### 4.2. DNA Isolation and Sequencing

DNA isolation and sequencing of the *ethA, inhA* and P*_fabG1_* fragments were performed as previously described [48]. The following PCR primers were used: *ethAR*-F1: 5′-cgacgttgaaatcacgctgg-3′, *ethAR*-R1: 5′-gtgaccgacaccattgaacg-3′; *ethAR*-F2: 5′-ttcaaccccgt-tgcggtaat-3′; *ethAR*-R2: 5′-ctctttctgtgcagcggcta-3′; *ethAR*-F3: 5′-atgatcggcccgacgaaatc-3′; *ethAR*-R3: 5′-ccctggcagcttactacgtg-3′; P*_fabG1_*-F: 5′-cctcgctgcccagaaaggga-3′; P*_fabG1_*-R: 5′-atcc-cccggtttcctccggt-3′-3′; *inhA*-F2: 5′-gagctatatctccggtgcgg-3′; *inhA*-R2: 5′-gcgaccgtcatcca-gttgta-3′; *inhA*-F3: 5′-ccacatctcggcgtattcgt-3′; *inhA*-R3: 5′-cggtgataccccaccgaaat-3′.

## 5. Conclusions

Phenotypic methods for testing ethionamide susceptibility using liquid media are poorly correlated. The isolates with MIC = 5 mg/L obtained using the Sensititre microtiter assay should be treated as isolates with intermediate resistance. A significant proportion of MDR and XDR cases are susceptible and eligible for treatment with ethionamide. The promoter mutation P*_fabG1_* c(-15)t predicts resistance to ethionamide with high specificity, but low sensitivity. Resistance could be explained using only the analysis of the loci *ethA*, P*_fabG1_*, and *inhA* for most isolates, which circulate in the Moscow region.

## Figures and Tables

**Table 1:** Number of isolates with different resistance profiles.

Drug Resistance Profile	MIC Ethionamide, mg/L									Isolates, Total
	0.3	0.6	1.25	2.5	5	10	20	40	>40	
Eth-S *	3	17	45	63	37	10	1			176
Eth-R *	1		7	28	38	57	20	13	9	173
S		4	22	24	3					53
mono/poly		1	11	18	13	5	4	3		55
MDR	1	5	8	17	12	12	3	2	3	63
pre-XDR	3	6	5	16	24	23	5	3		85
XDR		1	6	16	23	27	9	5	6	93

*—resistance to ethionamide (Eth) detected using Bactec MGIT 960 (critical concentration—5 mg/L).

**Table 2:** Comparison of pairwise MICs of ethionamide and isoniazid.

MIC Isoniazid, mg/L	MIC Ethionamide, mg/L									Isolates, Total
	0.3	0.6	1.25	2.5	5	10	20	40	>40	
0.03		3	7	2	2	1				15
0.06			17	27	3					47
0.13		1		2	1	1				5
0.25		1	1					1		3
0.5						1		1		2
1			2	2	1					5
2	1	6	4	12	8	10		1		42
4	1	3	9	18	23	15	4	2		75
>4	2	3	12	28	37	39	17	8	9	155
Isolates, total	4	17	52	91	75	67	21	13	9	349

**Table 3:** Molecular determinants of ethionamide resistance.

Mutation Profile	MIC Ethionamide, mg/L									Eth *	
	0.3	0.6	1.25	2.5	5	10	20	40	>40	S	R
wt	1	7	23	41	6	1				77	2
*ethA*_subst		1	3	4	4	2			1	8	7
*ethA*_fs			1	3	3	7	4		1	0	19
P*_ethA_*					1	1				0	2
P*_fabG1_*			1		1	3	2	1		3	5
*inhA*				3		1				2	2
P*_fabG1_* + *ethA*			1	1		1	2			0	4
P*_fabG1_* + *inhA*								1		0	1
*ethA* + *inhA*							1			0	1
P*_fabG1_* c(-15)t											
mutation			1	1		5	3	2		2	10
wt	1	8	28	51	15	14	6		2	88	37
All mutations											
mutation		1	6	11	9	17	9	2	2	13	44
wt	1	7	23	41	6	1				77	2
All mutations excluding *ethA* substitutions											
mutation			3	7	5	15	9	2	1	5	37
wt	1	8	26	45	10	3			1	85	9

*—resistance to ethionamide (Eth) detected using Bactec MGIT 960 (critical concentration—5 mg/L).

## Data Availability

Data are contained within the article or supplementary material.

## Supplementary Materials

The following are available online at https://www.mdpi.com/article/10.3390/antibiotics11020133/s1, Table S1: Isoniazid MIC for isolates with different resistance profiles, Table S2: Determinants of ethionamide resistance for 136 primary isolates.
