# Supplementary material for: Molecular Determinants of Ethionamide Resistance in Clinical Isolates of Mycobacterium tuberculosis
Source: Antibiotics (Basel). 2022 Jan 20;11(2):133. doi: 10.3390/antibiotics11020133 (PMC8868424; doi:10.3390/antibiotics11020133)
Supplement: Supplementary file 1 [file antibiotics-11-00133-s001.zip › antibiotics-1534312-supplementary.pdf]

antibiotics

Table S1. Isoniazid MIC for isolates with different resistance profiles.

Drug resistance profile	MIC isoniazid, mg/L									Isolates, total
	0.03	0.06	0.13	0.25	0.5	1	2	4	>4	
Inh-S*	15	47	2	2						66
Inh-R*			3	1	2	5	42	75	155	283
S	11	40	2							53
mono/poly	4	7	2	1	1		1	11	28	55
MDR				1		3	14	10	35	63
pre-XDR			1	1			13	16	54	85
XDR					1	2	14	38	38	93

* - resistance to isoniazid (Inh) detected using Bactec MGIT 960

Table S2. Determinants of ethionamide resistance for 136 primary isolates.

ID	Drug resistance profile	INH MGIT	INH MIC	ETH MGIT	ETH MIC	*ethA*	P_{fabG1}	*inhA*
1	MDR.Sl	R	5	R	10	wt	c(-15)t	wt
2	MDR.Sl	R	5	S	10	t713g V238G	wt	wt
3	MDR	R	5	S	10	wt	wt	wt
4	poly	R	5	S	10	wt	c(-15)t	wt
5	poly	R	5	R	10	wt	c(-15)t	wt
6	MDR.Km	R	5	R	10	t(-7)c	c(-15)t	c707t T236M
7	poly	R	5	R	10	c1022t A341V	c(-15)t	wt
8	poly	R	5	R	20	g63gc	wt	wt
9	poly	R	5	R	20	wt	c(-15)t	wt
10	poly	R	5	R	20	wt	c(-15)t	wt
11	poly	R	5	R	40	wt	c(-15)t	wt
12	poly	R	4	R	1.2	ca1341c	c(-34)t	wt
13	poly	R	5	R	1.2	ga598g	wt	wt
14	poly	R	5	S	1.2	wt	c(-15)t	wt
15	poly	S	0.25	S	1.2	wt	wt	wt
16	MDR	R	5	R	2.5	wt	wt	wt
17	MDR	R	5	R	2.5	wt	wt	wt
18	poly	R	5	R	2.5	t904c F302L	c(-15)t	wt
19	MDR	R	5	S	2.5	wt	wt	wt
20	MDR	R	5	S	2.5	wt	wt	wt
21	MDR	R	5	S	2.5	wt	wt	wt
22	MDR	R	5	S	2.5	wt	wt	wt
23	MDR	R	5	S	2.5	wt	wt	wt
24	MDR.Sl	R	4	R	5	g789c M263I	wt	wt

25	MDR.Fq	R	5	R	5	c941t T314I	wt	wt
26	poly	R	5	R	5	t1319c L440P	wt	wt
27	MDR	R	5	S	5	wt	wt	wt
28	MDR.Sl	R	5	S	5	c941t T314I	wt	wt
29	MDR.Fq	R	5	S	5	wt	wt	wt
30	MDR	R	5	S	5	wt	wt	wt
31	poly	S	0.25	S	0.6	wt	wt	wt
32	MDR	R	5	S	0.3	wt	wt	wt
33	MDR.Sl	R	5	R	10	wt	wt	g38a S13L; t347c V116A
34	S	S	0.06	S	1.2	wt	wt	wt
35	S	R	4	S	1.2	wt	wt	wt
36	S	S	0.06	S	2.5	wt	wt	wt
37	S	S	0.06	S	1.2	wt	wt	wt
38	S	R	4	S	1.2	wt	wt	wt
39	S	R	4	S	2.5	wt	wt	wt
40	S	R	5	S	2.5	wt	wt	wt
41	MDR.Fq	R	5	S	2.5	wt	wt	wt
42	S	S	0.06	S	2.5	wt	wt	wt
43	MDR	R	5	R	10	ga337g	wt	wt
44	S	S	0.06	S	2.5	wt	wt	wt
45	MDR.Fq	R	5	R	20	c1243ca	c(-15)t	wt
46	S	S	0.06	S	2.5	wt	wt	wt
47	S	S	0.03	R	10	t68g L23R	wt	wt
48	S	S	0.06	S	2.5	wt	wt	wt
49	MDR.Sl	R	5	R	10	c862a P288T; ga106g	wt	wt
50	MDR	R	5	R	20	c745ca	wt	wt
51	MDR.Sl	R	5	R	10	ga337g	wt	wt
52	S	R	4	R	5	g1173a W391*	wt	wt
53	S	S	0.06	S	1.2	wt	wt	wt
54	S	S	0.06	S	1.2	c390t T130T	wt	wt
55	XDR	R	4	R	5	at1009a	wt	wt
56	S	S	0.06	S	1.2	wt	wt	wt
57	S	S	0.06	S	1.2	wt	wt	wt
58	MDR.Sl	R	4	R	10	t(-6)c	wt	wt
59	S	S	0.03	S	2.5	wt	wt	wt
60	MDR	R	2	S	0.6	t1319c L440P	wt	wt
61	MDR	R	1	S	2.5	wt	wt	wt
62	S	R	5	S	2.5	wt	wt	wt
63	MDR	R	2	R	40	wt	c(-15)t	t589c I194V
64	MDR	R	4	R	10	wt	g(-55)a	wt

65	S	S	0.06	S	2.5	wt	wt	wt
66	MDR	R	2	R	5	ct106c	wt	wt
67	S	S	0.06	S	2.5	wt	wt	wt
68	MDR.Sl	R	4	S	0.6	wt	wt	wt
69	S	S	0.06	S	2.5	wt	wt	wt
70	MDR	R	5	S	1.2	wt	wt	wt
71	MDR	R	4	R	2.5	a749t Y250F, t746tg	wt	wt
72	MDR	R	2	R	2.5	c329g S110W	wt	wt
73	MDR	R	2	S	0.6	wt	wt	wt
74	S	S	0.06	S	2.5	wt	wt	g291a F97L
75	S	R	5	S	2.5	wt	wt	wt
76	S	S	0.03	S	1.2	wt	wt	wt
77	S	S	0.06	S	1.2	wt	wt	wt
78	S	S	0.06	S	2.5	c64t H22Y	wt	wt
79	S	S	0.06	S	1.2	a869g D290G	wt	wt
80	S	S	0.06	S	1.2	a697t M233L	wt	wt
81	S	R	5	S	5	wt	wt	wt
82	MDR	R	5	R	41	g352c V118L, a632g T211C	wt	wt
83	MDR	R	4	R	2.5	wt	wt	t739c S247P
84	MDR	R	5	R	41	t276tggaa	wt	wt
85	S	S	0.06	S	2.5	wt	wt	wt
86	S	S	0.06	S	1.2	wt	wt	wt
87	MDR	R	5	R	20	ac941c	wt	wt
88	S	S	0.06	S	2.5	wt	wt	wt
89	S	S	0.06	S	1.2	wt	wt	wt
90	S	S	0.06	S	1.2	wt	wt	wt
91	S	R	5	S	2.5	wt	wt	wt
92	XDR	R	4	R	20	ga106g	wt	wt
93	S	S	0.03	S	0.6	wt	wt	wt
94	S	S	0.03	S	0.6	wt	wt	wt
95	S	S	0.125	S	5	wt	t(-8)c	wt
96	MDR.Sl	R	5	R	10	ga337g	c(-34)t	wt
97	S	S	0.125	S	2.5	wt	wt	wt
98	S	S	0.06	S	1.2	wt	wt	wt
99	MDR.Sl	R	4	S	2.5	wt	wt	wt
100	S	S	0.06	S	2.5	wt	wt	wt
101	XDR	R	4	R	5	t(-7)c	wt	wt
102	S	S	0.03	S	1.2	wt	wt	wt
103	S	R	0.125	S	2.5	wt	wt	wt
104	MDR.Sl	R	5	R	10	t(-7)c	wt	g707a T236K

105	S	S	0.06	S	2.5	wt	wt	wt
106	XDR	R	5	R	10	ga106g	wt	wt
107	S	S	0.06	S	2.5	c15t L5L	wt	wt
108	S	S	0.06	S	2.5	wt	wt	wt
109	S	R	5	S	2.5	wt	wt	wt
110	S	R	4	S	1.2	wt	wt	wt
111	MDR	R	4	S	1.2	t1319c L440P	wt	wt
112	S	S	0.06	S	2.5	wt	wt	wt
113	S	R	4	S	2.5	wt	wt	wt
114	MDR.Sl	R	5	R	10	t276a Y92*, a279g G93G	wt	wt
115	MDR.Sl	R	5	R	20	t276tggaa	t(-8)c	wt
116	S	S	0.06	S	2.5	wt	wt	wt
117	S	S	0.06	S	1.2	wt	wt	wt
118	S	S	0.06	S	1.2	c231t D77D	wt	wt
119	MDR	R	4	R	10	tc150t, c652a R218R	wt	wt
120	S	S	0.06	S	1.2	wt	wt	wt
121	S	S	0.03	S	1.2	wt	wt	wt
122	S	S	0.03	S	1.2	wt	wt	wt
123	MDR	R	5	R	2.5	c941t T314I	wt	wt
124	S	S	0.03	S	0.6	wt	wt	wt
125	MDR	R	4	R	2.5	ct882c	wt	wt
126	MDR.Sl	R	4	R	2.5	ct882c	wt	wt
127	MDR.Sl	R	5	S	2.5	wt	wt	wt
128	S	S	0.06	S	2.5	wt	wt	wt
129	S	R	5	S	5	wt	wt	wt
130	MDR.Sl	R	5	R	20	ct702c	wt	g535a A179T
131	S	R	4	S	2.5	wt	wt	wt
132	MDR	R	2	S	0.6	wt	wt	wt
133	S	S	0.06	S	2.5	c231t D77E, g1338a L446L	wt	wt
134	S	S	0.06	S	5	wt	wt	wt
135	S	R	5	S	2.5	wt	wt	wt
136	S	S	0.06	S	2.5	wt	wt	wt
137	S	S	0.06	S	2.5	wt	wt	g291a F97L
